# Maintenance of Physical Activity Behavior by Individuals with Prediabetes and Diabetes during the COVID-19 Pandemic after Completing an Exercise Intervention in Brazil

**DOI:** 10.3390/ijerph19148857

**Published:** 2022-07-21

**Authors:** Isabela Coelho Ponciano, Mariana Balbi Seixas, Tiago Peçanha, Adriano Luiz Pereira, Patrícia Fernandes Trevizan, Raquel Rodrigues Britto, Lilian Pinto da Silva

**Affiliations:** 1 Graduate Program in Rehabilitation Sciences and Physical-Functional Performance, Faculty of Physical Therapy, Federal University of Juiz de Fora, Avenida Eugênio do Nascimento S/N, Juiz de Fora 36038-330, MG, Brazil; isabelacponciano@gmail.com (I.C.P.); t.pecanha@mmu.ac.uk (T.P.); adrianolp77@hotmail.com (A.L.P.); r3britto@gmail.com (R.R.B.); 2 Cardiovascular Research Unit and Exercise Physiology, University Hospital, Federal University of Juiz de Fora, Avenida Eugênio do Nascimento S/N, Juiz de Fora 36038-330, MG, Brazil; mariana.balbi@ufjf.br (M.B.S.); patricia_trevizan@yahoo.com.br (P.F.T.); 3 Graduate Program in Physical Education, Faculty of Physical Education and Sports, Federal University of Juiz de Fora, Rua José Lourenço Kelmer S/N, Juiz de Fora 36036-900, MG, Brazil; 4 Department of Sport and Exercise Sciences, Musculoskeletal Science and Sports Medicine Research Centre, Faculty of Science & Engineering, Manchester Metropolitan University, 99 Oxford Road, Manchester M1 7EL, UK; 5 Department of Physical Therapy, Federal University of Minas Gerais, Avenida Presidente Antônio Carlos, 6627-Pampulha, Belo Horizonte 31270-901, MG, Brazil

**Keywords:** diabetes mellitus, physical activity, maintenance, perception, adherence, motivation

## Abstract

This cross-sectional study evaluated the perception of individuals with prediabetes/diabetes about their living conditions during the COVID-19 pandemic to identify the facilitators, barriers, and reasons to remain physically active at home and adhere to recommended exercise. It included individuals with prediabetes/diabetes who had completed an exercise intervention, which started on-site and moved to a remote home-based regime due to the COVID-19 pandemic and were advised to keep exercising at home. The outcomes were assessed by a bespoke questionnaire that was developed by the research team, the Brazilian Portuguese adapted version of the Exercise Adherence Rating scale, and the Motives for Physical Activity Measure-Revised scale. Of 15 participants (8 female, 58 ± 11 years), most reported positive perceptions about their living conditions and few difficulties maintaining some physical activity at home. However, only 53.8% of them adhered to the recommended exercise. Time flexibility, no need for commuting, and a sense of autonomy were the main facilitators of home exercise, while a lack of adequate space was the main barrier. The descending order of median scores that were obtained in each reason for physical activity was fitness, enjoyment, competence, social, and appearance. Individuals with prediabetes/diabetes maintained some physical activity during the pandemic, mainly motivated by health concerns.

## 1. Introduction

As a chronic disease that requires continuous care for glycemic control and the prevention of its complications, diabetes care demands different approaches [1], including routine physical activity [2]. Physical exercise contributes to glycemic control, weight loss, positive self-perception of health status, improved cardiorespiratory fitness, well-being, and quality of life in individuals living with diabetes [2,3,4]. Additionally, physical activity promotes Type 2 diabetes prevention in individuals with prediabetes [3,5]. Supervised and structured exercise programs promote these benefits more efficiently than non-supervised ones [6]. However, studies have shown a progressive decrease in exercise adherence after the completion of these programs [3,7]. This reduction in exercise adherence is possibly caused by barriers such as a lack of understanding of exercise instructions that are provided by health professionals; difficulty fitting exercise into a daily routine; hypoglycemia concern during exercise, especially in people with Type 1 diabetes [8,9]; and a lack of pleasure and motivation to exercise [7].

The COVID-19 pandemic [8] imposed significant social changes that affected people’s lifestyles and behaviors [9,10]. During the lockdown, people with Type 2 diabetes increased sitting time and decreased minutes of walking or other moderate physical activity per week [11]. At the same time, healthcare systems (e.g., NHS) [12], medical societies [13], and researchers [14] recommended home-based exercises to maintain population physical activity levels and to address the physical and mental health problems that were caused or worsened by the pandemic [15,16].

Indeed, there is evidence that home-based exercises can improve muscle strength, functional capacity, and quality of life in patients with autoimmune [17] and chronic diseases [18]. A recent systematic review demonstrated that home-based exercises are a safe and effective alternative to health management for people living with diabetes and prediabetes [19]. However, adherence to home-based exercise is usually low [20], evidencing that exercise maintenance is complex, as it does not depend only on guidance and encouragement from healthcare professionals.

This study aims to assess individuals with prediabetes and diabetes that had to move from a structured and on-site to a remote home-exercise intervention because of the COVID-19 pandemic to (a) evaluate their perception of their living conditions during the COVID-19 pandemic; (b) identify facilitators, barriers, and motives to remain physically active at home during the pandemic; and (c) investigate their adherence to exercise as recommended when completed the exercise intervention.

## 2. Materials and Methods

This descriptive cross-sectional study was approved by the Research Ethics Committee of the Hospital of Federal University of Juiz de Fora (CAAE: 36267420.3.0000.5133) and involved a convenience sample of participants who completed the exercise intervention of the pilot study of Diabetes College Brazil trial (NCT03914924). In that pilot study, individuals with prediabetes and diabetes participated in a 12-week exercise intervention lasting. The inclusion criteria were as follows: age ≥ 18 years, clinical diagnosis of prediabetes (fasting glucose ≥ 100 and <126 mg/dL or glycated hemoglobin ≥ 5.7 e < 6.5%) [21] or diabetes (fasting glucose >126 mg/dL or glycated hemoglobin > 6.5%) [21]; no cognitive limitation (i.e., six-item screener score ≥ 4) [22]; no confirmed diagnosis of unstable coronary artery disease or heart failure; no pacemaker and/or implantable cardioverter-defibrillator; no intermittent claudication; no recent cardiovascular event or cardiac surgery (≤6 months); and not enrolled currently in a structured physical exercise program that follows diabetes guidelines. The exclusion criteria were: clinical decompensation that contraindicated physical exercising, physical and/or mental limitations that prevented the participant from physical exercise and/or understanding educational content, and complex ventricular arrhythmias. Due to the social restrictions that were imposed by the COVID-19 pandemic, it was impossible to carry out all the supervised on-site exercise sessions as planned. Therefore, the intervention was adapted to be delivered remotely through weekly phone calls and a video with home-based exercises recorded by the research team [23]. All the individuals who completed participation in the pilot study were instructed to maintain at least 150 min of moderate- or vigorous-intensity aerobic physical activity and 2 to 3 sessions/week of resistance exercise, as recommended by the diabetes guidelines [1,21,24], and were invited to participate in the present study.

The data collection started in August 2020, three months after completing the exercise intervention. The individuals were contacted over a maximum of three phone call attempts to receive the invitation to participate in the present study. The study details were presented, and the individuals who agreed to participate received, via WhatsApp^®^, a Google^®^ form containing the study consent form and the three questionnaires that are described below. All the participants signed the online consent form before being included in the study. Their sociodemographic and clinical characteristics were obtained from previously collected data in the Diabetes College Brazil trial pilot baseline (submitted data).

The perceptions about living conditions during the pandemic and the facilitators and barriers to maintaining physical activity at home were assessed by a bespoke 8-item questionnaire that was developed exclusively for this purpose by the research team (Appendix A). Perceptions regarding living conditions during the pandemic were evaluated based on the responses to items 1 to 5 of this questionnaire, and facilitators and barriers to exercising at home were assessed based on items 6 to 8 responses.

The motives for remaining physically active were evaluated by the Motives for Physical Activity Measure-Revised scale (MPAM-R) [25,26]. This scale is a self-administered questionnaire that contains twenty-six items that encompass five general motives: enjoyment (seven items), competence (four items), appearance (six items), fitness (four items), and social (five items). Each item should be responded to on a 7-point Likert scale (1—“not at all true for me” to 7—“very true for me”). This questionnaire is based on the Self-Determination Theory [27], which has been used to understand the motivation for physical activity in different populations [28]. Among the motives for physical activity that were assessed by the MPAM-R scale, enjoyment and competence are related to intrinsic motivation, and the others refer to extrinsic motivation.

Exercise adherence, as recommended, was evaluated by the Brazilian Portuguese version of the Exercise Adherence Rating scale (EARS-Br) [29,30]. This scale is a self-administered questionnaire that contains six items that are scored by an ordinal answer range (0 = strongly agree to 4 = totally disagree) ranging from 0 to 24, and a score of seventeen points is a cut-off point that demarks adequate adherence to the recommended exercise [30].

Categorical data were analyzed by calculating simple frequencies and percentiles. The normal distribution of numerical data was tested using the Shapiro–Wilk test, adopting a significance level of 5%. Variables with normal distribution were expressed as the mean ± standard deviation, while those with non-normal distribution were expressed as the median and interquartile range. IBM SPSS Statistics, v. 26, software was used for data analysis.

## 3. Results

A total of 33 individuals were eligible to participate in the study, of which 16 answered the research team phone call, and 15 agreed to participate. All the participants completed the three questionnaires online. The clinical and sociodemographic characteristics of the participants are described in Table 1.

Most participants reported dealing well with the fact that COVID-19 disease is highly prevalent in individuals with diabetes and that it is associated with increased incidence of disease severity and mortality. Regarding other aspects of living conditions during the pandemic, the higher response rate was in options that express positive perceptions, as described in Table 2.

A total of 13 participants (87%) reported having managed to maintain physical activity during the pandemic (item 6 of Appendix A). The affirmatives presenting exercise barriers (item 7 of Appendix A) had an agreement response rate lower than 50%. The lack of adequate space was the most significant barrier (40%) to maintaining physical activity at home. The affirmatives presenting exercise facilitators (item 7 of Appendix A) had an agreement response rate that was higher than 50%, and the flexibility of time, no need for commuting, and the sense of autonomy were pointed out as the main facilitators, as described in Table 3. Most participants (73%, n = 11) reported that they would choose supervised on-site exercise sessions if they had this possibility (item 8 of Appendix A).

### 3.1. Motives for Physical Activity Maintenance

**Figure 1:**
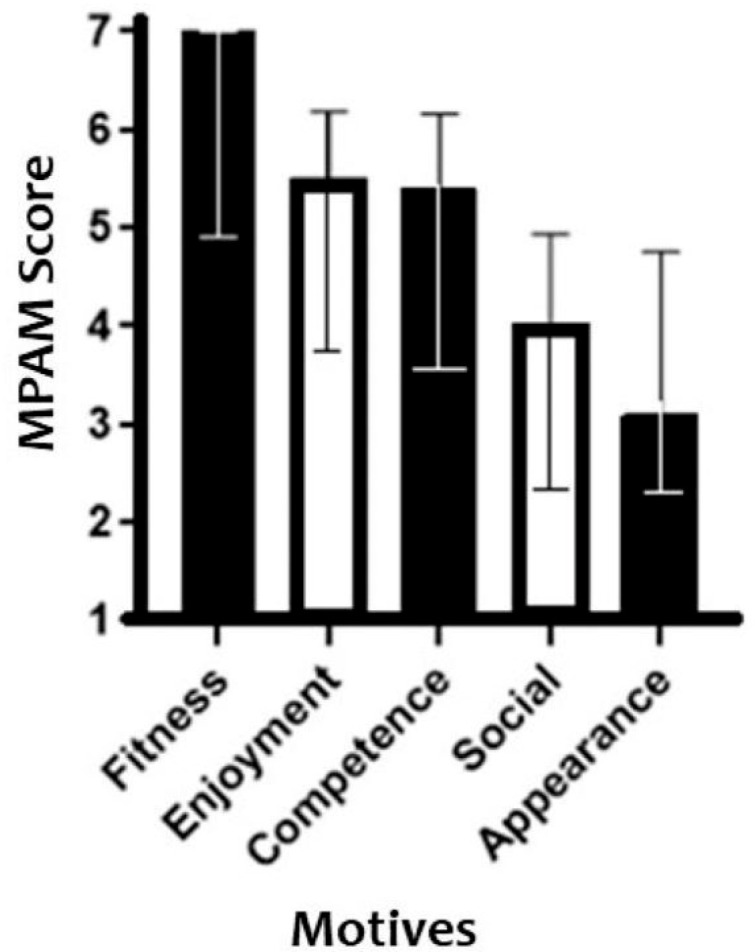
Scores to each MPAM-R item. Values are expressed as the median and interquartile ranges.

Among the five motives for physical activity that were evaluated by the MPAM-R, fitness achieved the highest score while appearance had the lowest, as shown in Figure 1.

### 3.2. Exercise Adherence

The median (interquartile range) of the EARS-Br total score was 17 (13–23), consequently revealing that 53.3% (n = 8) of the participants adhered, and 46.7% (n = 7) did not comply with the exercise according to recommendations that were received. Table 4 describes the response rate that was obtained for each EARS-Br scale response option.

## 4. Discussion

This study observed that the individuals with prediabetes and diabetes moving from an on-site supervised to a remote home-based exercise intervention because of the COVID-19 pandemic maintained some physical activity at home three months after this intervention, motivated by health concerns. Additionally, most participants had positive perceptions about their living conditions during the pandemic. Over half of them adhered to aerobic physical activity and resistance exercise as recommended at the end of the exercise intervention.

Most participants reported positive perceptions about their living conditions during the COVID-19 pandemic. Sociodemographic characteristics, such as household income and an educational level that was higher than observed in most of the Brazilian population (average household income: 2 minimum wages received monthly; educational level: 41% highschool concluded or higher) [31], may have positively influenced the participants’ perception of their living conditions in the present study. In addition, most participants were married, did not live alone, and were employed or retired, which possibly provided social, emotional, and financial support during the pandemic. The period in which the data were collected may also help to explain the positive perceptions since there were reduced social restrictions that were imposed by the pandemic in the city where the study was conducted at the data collection time [32]. In fact, a previous study [33] revealed that lifting the social restrictions that were imposed by the pandemic contributed to improving the quality of life of individuals with diabetes. Another possible explanation for the positive perceptions during the pandemic was maintaining some level of physical activity, which has been associated with positive psychological well-being [34]. Indeed, most participants reported physical activity maintenance during the pandemic. The main facilitators of home-based exercise were time flexibility, no need for commuting, and a sense of autonomy.

The results from exercise adherence revealed that only 53.3% of the participants fully complied with the exercise as recommended after an exercise intervention. The lack of exercise adherence that was scored by 46.7% of the participants may have been influenced by the lack of adequate space and insecurity to perform them without supervision, considering that these statements had a higher percentage of agreement than the other barriers to exercising at home. In addition, the age of the participants may have contributed to this finding since there is a decrease in willingness to exercise with aging as exercise self-efficacy is negatively correlated with age [35]. Approximately three-quarters of the study participants had Type 2 diabetes or pre-diabetes, conditions that are more prevalent in the older age group [21,36]. Even if some participants did not fully comply with exercise as recommended by the diabetes guidelines [1,21,24], it is important to recognize that they managed to maintain some level of physical activity. This finding corroborates the recent view that was portrayed by current Physical Activity Guidelines [37], which considers that “Every Move Counts” and “doing some physical activity is better than doing nothing”.

Fitness was pointed out as the primary motive for maintaining physical activity at home during the pandemic. Fitness is considered an extrinsic motivation since the individual’s behavior is stimulated by the appreciation of the results and benefits of participation in a given activity, disease prevention, and treatment or physical condition improvement [28]. This finding disagrees with studies [34,38] that were also carried out during the pandemic, which found intrinsic factors such as pleasure as the main motive for continued exercise. However, these studies were carried out with healthy adults, possibly explaining why they did not find “fitness” as their primary motive for exercising. On the other hand, another study [39] showed mental health, another extrinsic motivation, as being both a barrier and a motivator for physical exercise at the same time. During stressful periods such as the pandemic, people are primarily motivated to be physically active to manage stress levels and anxiety and improve sleep [39]. However, they may also be too anxious or depressed to start and maintain physical exercise [39]. The “social” motive is an extrinsic motivation that has been pointed out in other studies [40] as an important motivating factor to maintain physical activity as the presence of other people working at a similar activity not only creates a sense of shared identity but is also a source of healthy competition and hence motivation. The few barriers that were perceived by the participants to home-based physical activity engagement possibly contributed to the “social” motive being poorly scored in this study. On the other hand, this motive could be indirectly related to the preference of most participants for supervised and on-site exercise sessions.

This is the first study to investigate the psychosocial aspects and living conditions, facilitators, and barriers to home-based physical activity; motives for physical activity; and adherence to aerobic physical activity and resistance exercise as recommended by diabetes guidelines [1,21,24] in individuals with prediabetes and diabetes during the COVID-19 pandemic. These behavioral and emotional factors interfere with blood glucose levels [40] and adhesion to exercise [41]; therefore, the understanding of factors can support health professionals to adapt the recommendations individually to achieve better exercise adherence.

The study limitations are the small number of participants and the fact that they have experienced an exercise intervention. As such, it is not possible to generalize the results to the population with prediabetes and diabetes who did not have this previous experience. In addition, the comparison with studies outside the pandemic context is limited, and, as it is a cross-sectional study, it is not possible to assume a causal relationship from its results. Despite these limitations, the study indicates perceptions that are related to the maintenance of physical exercises at home that can be considered in future research as well as in prescribing home-based exercise to this population.

## 5. Conclusions

Most participants in this study dealt well with their health condition during the pandemic and reported few difficulties maintaining their physical activity at home, mainly motivated by healthcare concerns. The lack of adequate space was the most significant barrier to home-based exercising. Time flexibility, no need for commuting, and a sense of autonomy were the main facilitators of physical activity maintenance.

## Figures and Tables

**Table 1:** Participant’s sociodemographic and clinical characteristics.

Variables		(n = 15)
Sex	Female	53.3% (8)
Age		58 ± 11 years
Body mass index		27.5 ± 3.2 kg/m^2^
Diabetes type	Prediabetes	13.3% (2)
	Type 1	26.7% (4)
	Type 2	60% (9)
Glycated hemoglobin ^a^		7.8 ± 1.8%
Insulin Therapy		40% (6)
Time elapsed since diagnosis		9 ± 7 years
Comorbidities	Hypertension	46.6% (7)
	Dyslipidemia	46.6% (7)
	Stroke	12.5% (2)
	Coronary arterial disease	12.5% (2)
Educational level	Elementary school concluded	13.3% (2)
	High-school concluded	53.3% (8)
	Undergraduate concluded	33.3% (5)
Household income ^b^	≤2	13.3% (2)
	>2 up to 6	60% (9)
	>6	26.7% (4)
Work status	Employed	53.3% (8)
	Unemployed	6.7% (1)
	Retired	26.7% (4)
	Homemaker	13.3% (2)
Healthcare funding	Public	20% (3)
	Private	80% (12)
Marital status	Single	26.7% (4)
	Married	60% (9)
	Widow	6.7% (1)
	Divorced	6.7% (1)
	Living alone	26.6% (4)

Values are expressed as the mean ± standard deviation or percentages (number of participants). ^a^ n = 10. ^b^ Household income was reported as the number of Brazilian minimum wages received monthly and paid in reais.

**Table 2:** Response rate to each answer option of questions that were related to the living condition perceptions during the pandemic.

Items	Response Options	Response Rate
Question 1:		
In general, what have been your most predominant feelings in this period of social restrictions?	Calm, well, peaceful, patient, animated, hopeful.	66.7% (10)
	Depressed, worried, tired, impatient, inactive.	33.3% (5)
	Happy, confident, excited, energetic, active, enthusiastic.	20% (3)
	Unhappy, scared, afraid, incapable, alone.	13.3% (2)
	Upset, angry, agitated, exhausted.	0% (0)
Question 2:		
Knowing that COVID-19 is highly prevalent in patients with diabetes and associated with increased incidence of disease severity and mortality, did you handle this situation well?	Yes	80% (12)
	No	20% (3)
Question 3:		
Choose the alternative that you most identify concerning being a person with prediabetes or diabetes going through the COVID-19 pandemic.	I have been coping well and have continued following main preventive measures (ex.: washing hands and masking).	46.7% (7)
	I have been coping well, but I am worried, and I have isolated myself as much as possible from people and interrupted some activities.	40% (6)
	I am afraid of getting the virus. So, I isolated myself from my family and interrupted my activities, staying at home. I am feeling well this way.	6.7% (1)
	I am afraid of getting the virus. So, I isolated myself from my family and suspended my activities, staying at home. I am feeling depressed this way.	6.7% (1)
	I am petrified and apprehensive about getting the virus and worsening my health. I feel lonely, sad, and hopeless.	6.7% (1)
Question 4:		
In general, what is the impact of the COVID 19 pandemic on your life?		
Psychological aspects	It has been a good time to review and reorganize life, and overall, I have been coping well and enjoying this time.	33.3% (5)
	I was not affected much because my routine hadn’t changed.	20% (3)
	In the beginning, it affected me a lot, but now I have adapted, and I can take control of my feelings.	20% (3)
	It has been difficult, and it has affected my mood, my feelings a lot… I am depressed most of the time.	12.5% (2)
	It negatively affected the psychological a lot, and I don’t know how to deal with my feelings. I had crises of anxiety, depression, feelings of fear, loneliness…	0% (0)
	Other *“As I started working recently and I need to leave my home. I had moments of anxiety, depression, fear, and loneliness. I’m mostly afraid because I perceive that many people don’t seem to care about the situation we are facing now. I think I’m just scared…”* *“Public transportation worries me.”* *“I worry a lot about contamination risk as I see that people don’t protect themselves. I keep going to work. My diabetes is under control. I am not walking at the moment, but I do strengthening and stretching exercises almost daily.”*	20% (3)
Family life aspects	I was not affected much because my family life remained as it was before.	33.3% (5)
	It has been a good time of union, good family life, and greater empathy.	26.7% (4)
	I am afraid of getting the virus from some family member or putting my family at risk, so I isolated myself from them.	20% (3)
	It has not been easy to live together with family and accommodate housework and working at home simultaneously.	13.3% (2)
	It has been difficult due to the children or elderly or sick family members I have to take care of.	0% (0)
	Other *“It has been exhausting to do housework as I live in a big house with my husband and children. In addition, we all have been indoors 24/7 for five months.”*	6.7% (1)
Financial aspects	There was no change regarding my finances.	31.3% (5)
	My income decreased, but I am handling it well, and I am still employed.	26.7% (4)
	I lost my job, but I am handling it well.	13.3% (2)
	I lost my job, and I am not handling it well and need financial support from others.	0% (0)
	It’s been challenging financially.	0% (0)
	Other *“I started to work but have not been paid yet, and it worries me.”* *“Income has reduced a lot; we had to stop paying some bills and cut several expenses, including firing our maid who had been working at our home for five years.”*	12.5% (2)
Health condition aspects	My health condition improved.	47.1% (8)
	Participant quotes: “*My care is doubled.”* *“I am taking better care of myself.”* *“Better care and prevention.”* *“I am controlling my diabetes.”* *“I improved the frequency of physical activities; I started to practice mountain biking 3 to 4 times a week.”* *“I improved my fitness; walking and exercising.”* *“I implemented sanitary procedures in both the home and work environment. I started eating better to boost my immune system. I decided to take better care of my condition as a person living with diabetes.”*	
	My health condition remained as it was before.	23.5% (4)
	My health condition worsened; I had uncontrolled blood glucose, difficulties accessing the doctor and medicines.	11.8% (2)
	Some things have changed, such as sleep problems; I haven’t been able to eat well and take my medication as prescribed.	0% (0)
	My health condition and my blood glucose control worsened a lot, and, additionally, I had other complications imposing immediate medical care.	0% (0)
	Other *“Treatments in gynecology and gastrology were suspended, and I am waiting for a surgery.”* *“Some things have changed in my life right now. I have problems sleeping at night; I haven’t been eating well and taking my medication as prescribed.”* *“My blood glucose has been remaining under control during the pandemic with insulin use, diet, and walking, but I am having health problems such as shoulder and neck pain and numbness in my hands, which I relate to sleepless nights, tension, anxiety. In addition, the supervised functional exercise sessions that I was attending were suspended.”*	17.6% (3)
Question 5: Would you like to express something more about your perception of how you have felt in this period of social distancing?	*“Public transport is horrible.”* *“I miss the freedom to get together with friends.”* *“Kind of sad with the world situation in public health, social policy… Personally, I was not affected financially, and I didn’t even lose contact with family and friends as we have talked and seen each other virtually. I just miss being more in contact with nature that I enjoy a lot, as well as dancing and leisure in general.”* *“I have been working from home, and I only leave to go to the market, missing out on doing outdoor activities.”* *“Distance from family members, who most need my attention, as my mother of 91 who lives far away.”* *“Very bad experience not being able to be with the family for so long, even living in the same city. I have many brothers, nephews, and friends, and we see each other often. Spending all day in fear of someone in the family being infected and dying of COVID19 is terrifying.”* *“During this period, I have to adapt my work and exercise routine. I have been doing more resistance exercises because I spend more time at home.”* *“Communicating more with family and friends over the phone. We have been bonding even more now due to the use of phone and WhatsApp frequently.”*	

**Table 3:** Barriers and facilitators that were perceived by the participants to maintain exercising at home (n = 15).

Barriers	
Affirmatives	Agree or Totally Agree
It was difficult to keep up with the exercises because there was a lack of adequate space	40% (6)
It was difficult to maintain exercise because I was afraid or insecure about doing them by myself	26.7% (4)
The video I received with exercise options to replace walking was not explanatory enough	20% (3)
I did not keep up with the exercises due to a lack of time	20% (3)
I had problems related to my health that prevented me from exercising	13.3% (2)
I had personal problems that prevented me from exercising	13.3% (2)
**Facilitators**	
**Affirmatives**	**Agree or Totally Agree**
It was easier to exercise due to time flexibility, as I managed to organize my schedule better	73.3% (11)
It was easier because I did not have to go on-site	73.3% (11)
I felt more autonomy and independence to exercising	73.3% (11)
It was easier because I had more exercise options besides walking	66.7% (10)
My family was more involved with my exercises practicing, making it easier	53.4% (8)

Values are expressed as percentages (number of participants).

**Table 4:** Percentages of participants’ responses to each of the response options in the EARS-Br. (n = 15).

	Strongly Agree/Partially Agree	Totally Disagree/Partially Disagree	Neither Disagree nor Agree
**Items where agreeing indicates higher exercise adherence**			
I do my exercises as often as recommended	86.6% (13)	6.7% (1)	6.7% (1)
I fit my exercises into my regular routine	86.7% (13)	6.7% (1)	6.7% (1)
I do most, or all, of my exercises	80% (12)	13.4% (2)	6.7% (1)
**Items where agreeing indicates lower exercise adherence**			
I do less exercise than recommended by my healthcare professional	53.3% (8)	40.1% (6)	6.7% (1)
I don’t get around to doing my exercises	33.4% (5)	53.4% (8)	13.3% (2)
I forget to do my exercises	26.7% (4)	66.7% (10)	6.7% (1)

Values are expressed as percentages (number of participants).

## Data Availability

Not applicable.

## Appendix A

Questionnaire to assess the living condition perceptions during the pandemic and facilitators and barriers to maintaining home-based exercise

(1) In general, what have been your most predominant feelings in this period of social distancing? Note: It was possible to choose more than one answer option.
  ( ) Happy, confident, excited, energetic, active, enthusiastic
  ( ) Calm, well, peaceful, patient, animated, hopeful
  ( ) Depressed, worried, tired, impatient, inactive
  ( ) Unhappy, scared, afraid, incapable, alone
  ( ) Upset, angry, agitated, exhausted
(2) Knowing that COVID-19 is highly prevalent in patients with diabetes and associated with increased incidence of disease severity and mortality, did you handle this situation well? ( ) Yes ( ) No
(3) Choose the alternative that you most identify as concerning being a person with prediabetes or diabetes going through the COVID-19 pandemic:
  ( ) I have been coping well and have continued following main preventive measures (ex.: washing hands and masking)
  ( ) I have been coping well, but I am worried, have isolated myself as much as possible from people, and have interrupted some activities.
  ( ) I am afraid of getting the virus. So, I isolated myself from my family and interrupted my activities, staying at home. I am feeling well this way
  ( ) I am afraid of getting the virus. So, I isolated myself from my family and suspended my activities, staying at home. I am feeling depressed this way
  ( ) I am petrified and apprehensive about getting the virus and worsening my health. I feel lonely, sad, and hopeless
(4) In general, what is the impact of the COVID 19 pandemic on the following aspects of your life? Note: It was possible to choose more than one answer option.
  Psychological aspects
  ( ) I was not affected much because my routine hadn’t changed
  ( ) It has been a good time to review and reorganize life, and overall, I have been coping well and enjoying this time
  ( ) In the beginning, it affected me a lot, but now I have adapted, and I can take control of my feelings
  ( ) It has been difficult and affected my mood and my feelings a lot… I am depressed most of the time
  ( ) It negatively affected the psychological a lot, and I don’t know how to deal with my feelings. I had crises of anxiety, depression, feelings of fear, loneliness…
  ( ) Other: ________
  Family life aspects
  Do you live alone? ( ) Yes ( ) No
  ( ) I was not affected much because my family life remained as it was before
  ( ) It has been a good time of union, good family life, and greater empathy
  ( ) It has not been easy living together with family and having to accommodate housework and working at home at the same time
  ( ) It has been difficult due to the children or elderly or sick family members that I have to take care of.
  ( ) I am afraid of getting the virus from some family member or putting my family at risk, so I isolated myself from them
  ( ) Other: _______
  Financial aspects
  ( ) There was no change regarding my finances
  ( ) My income decreased, but I am handling it well, and I am still employed
  ( ) I lost my job, but I am handling it well
  ( ) I lost my job, and I am not handling it well and needed financial support from others
  ( ) It’s been challenging financially
  ( ) Other: _______
  Health condition aspects
  ( ) My health condition remained as it was before
  ( ) My health condition improved. If you have marked this item, please, describe what improvement(s) you have noticed in your health condition: _____________
  ( ) Some things have changed, such as I have been having problems sleeping at night, I haven’t been able to eat well and take my medication as prescribed
  ( ) My health condition worsened; I had uncontrolled blood glucose and difficulties accessing the doctor and medicines.
  ( ) My health condition and my blood glucose control worsened a lot, and, additionally, I had other complications imposing immediate medical care
  ( ) Other: _______
(5) Would you like to express something more about your perception of how you have felt in this period of social distancing? ____________________________________________
(6) In general, have you managed to maintain the practice of physical exercise in this period of the COVID-19 pandemic? ( ) Yes ( ) No
(7) Mark the option that reflects your perception of facilities and difficulties regarding the maintenance of physical exercise:

**Table A1:** Affirmatives concerning the facilities and difficulties for physical exercise maintenance.

	Totally Agree	Agree	Disagree	Totally Disagree	Not Know
It was easier to exercise due to time flexibility, as I managed to organize my schedule better	○	○	○	○	○
It was easier because I did not have to go on-site	○	○	○	○	○
I felt more autonomy and independence in exercising	○	○	○	○	○
My family was more involved with my exercise practicing, making it easier	○	○	○	○	○
It was easier because I had more exercise options besides walking	○	○	○	○	○
It was difficult to keep up with the exercises because there was a lack of adequate space	○	○	○	○	○
It was difficult to maintain exercise because I was afraid or insecure about doing them by myself	○	○	○	○	○
I had problems related to my health that prevented me from performing the exercise	○	○	○	○	○
I had personal problems that prevented me from performing the exercises	○	○	○	○	○
I did not keep up with the exercises due to a lack of time	○	○	○	○	○
The video I received with exercise options to replace walking was not explanatory enough	○	○	○	○	○

(8)If you could choose between performing supervised on-site exercises or home-based exercises, what would you choose?

( ) Supervised on-site exercises

( ) Home-based exercises

Why is that? _____________________________________________________________

_____________________________________________________________________

You have reached the end of this questionnaire! Thank you for your participation!
